# Two cases of thymoma with pulmonary metastasis: a case report

**DOI:** 10.1186/1477-7819-12-114

**Published:** 2014-04-23

**Authors:** Motoko Hirono, Makoto Nonaka, Naoya Himuro, Yuri Tomita, Daisuke Kataoka, Mitsutaka Kadokura

**Affiliations:** 1 Division of Chest Surgery, Department of Surgery, Showa University School of Medicine, 1-5-8 Hatanodai, 142-8555, Shinagawa-ku, Tokyo, Japan

**Keywords:** Thymoma, Pulmonary metastasis, Prognosis

## Abstract

**Background:**

Pulmonary metastases of thymomas are relatively rare. We report on two patients who underwent surgery for resection of pulmonary metastases.

**Methods and results:**

One patient was a 74-year-old man. A chest CT scan showed a mediastinal mass and a hilar nodule in the left lung. The patient underwent surgical resection of both of these lesions. The histological diagnosis was type A thymoma with intrapulmonary metastasis, classified as stage IVb. He did not receive any adjuvant therapy following the operation because the resection was complete. There has been no evidence of recurrence in four years.

The other patient was a 68-year-old man with myasthenia gravis. At the age of 61 years, he underwent extended thymectomy with combined resection of the surrounding involved structures. The histological diagnosis was type B3 thymoma, stage III. Adjuvant radiation (40 Gy) was administered postoperatively; however, a pulmonary nodule occurred seven years following the initial operation (patient age, 68 years). He subsequently underwent right lower lobectomy and a diagnosis of intrapulmonary metastasis of thymoma was made. There has been no evidence of recurrence in two years.

**Conclusions:**

Long-term follow-up is important to detect recurrence in any cases of thymoma. Lung metastases should be operated upon if they appear to be completely resectable and this can achieve long-term survival.

## Background

Thymomas are relatively rare mediastinal tumors arising from epithelial cells and accounting for approximately 0.2 to 1.5% of all malignancies [1]. Thymomas are typically indolent-growing tumors and have relatively good prognoses; however, their variable clinical patterns sometimes make it difficult to decide on optimum therapeutic strategy. We report on two patients who developed pulmonary metastasis with thymoma.

## Case presentation

**Figure 1:**
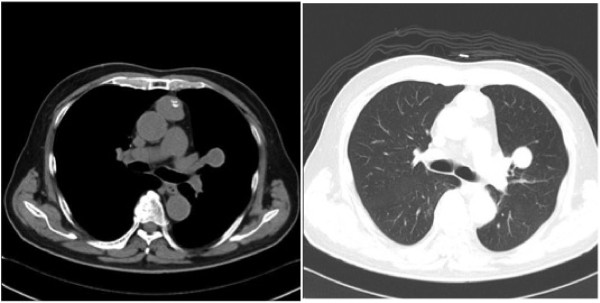
Computed tomography (CT) scan shows an anterior mediastinal mass with calcification, approximately 3 cm in diameter, and a 2 cm well-defined hilar nodule in the left upper lobe.

**Figure 2:**
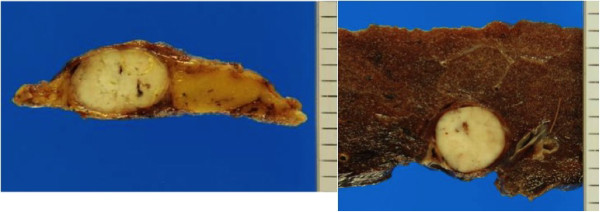
**Macroscopic finding of the pulmonary tumors.** The cut surface shows a completely encapsulated mass.

Case 1: the patient was a 74-year-old man without myasthenia gravis. An anterior mediastinal mass had been incidentally noticed on chest computed tomography (CT) approximately 10 years previously (patient age, 64 years). The patient refused surgery, and he was conservatively followed-up by annual CT scan. At the age of 74 years, a chest CT scan revealed a mediastinal mass, approximately 3 cm in diameter, and a 2 cm well-defined hilar nodule in the left upper lobe (Figure 1). The anterior mediastinal tumor was diagnosed as type A thymoma according to the World Health Organization (WHO) classification following percutaneous needle biopsy. Positron emission tomography (PET)-CT showed moderate ^18^ F-fluorodeoxyglucose (FDG) uptake in both lesions. We made a preoperative diagnosis of thymoma with intrapulmonary metastasis or thymoma combined with primary lung cancer. Therefore, thymo-thymectomy and left upper lobectomy were performed via a median sternotomy with transsternal extension. Both resected tumors were shown histologically to be type A thymoma (Figure 2), at stage IVb according to the Masaoka staging system [2]. The patient did not receive any postoperative adjuvant therapy because the resection had been complete. There has been no evidence of recurrence in four years.

**Figure 3:**
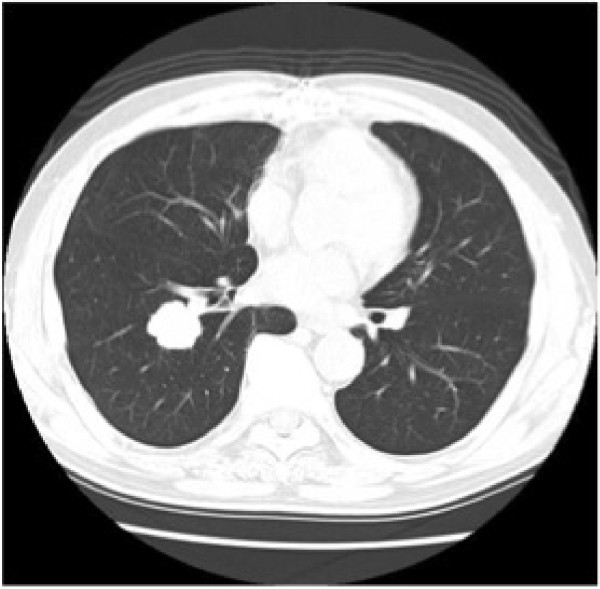
Another computed tomography (CT) scan shows a 2.5 cm well-defined nodule in the right lower lobe at seven years.

Case 2: the patient was a 68-year-old man with myasthenia gravis. He presented with invasive thymoma infiltrating into the right lung, pericardium, and right brachiocephalic vein at the age of 61 years. We performed extended thymectomy with combined resection. The tumor was diagnosed as type B3 thymoma according to the WHO classification. Histologic examination confirmed that the tumor was directly invasive only into the wall of the right brachiocephalic vein. The patient was shown to have stage III disease according to the Masaoka staging system. Adjuvant radiation (40 Gy) was performed postoperatively. A pulmonary nodule occurred seven years following the initial operation (patient age, 68 years) (Figure 3). PET-CT revealed moderate FDG uptake in the lesion of the right lower lobe. We diagnosed his disease preoperatively as pulmonary metastasis of invasive thymoma or second primary lung cancer. Therefore, right lower lobectomy was performed via right thoracotomy. The resected tumor was histologically the same as the former. He has been followed-up after the second operation, and there has been no evidence of recurrence at two years.

## Discussion

Several factors have been identified to influence the prognosis of thymomas after resection, such as clinical Masaoka stage, WHO histology, completeness of resection, and tumor size [3].

The first case in this report was cytologically benign, although the type A thymoma produced lung metastasis in the ten-year follow-up period. Generally, stage IVb thymoma is not an indication for surgery; however, surgery is recommended for a good prognosis if the tumor appears completely resectable [4]. Kondo and associates also demonstrated that adjuvant chemotherapy or radiotherapy did not improve the prognosis in patients with totally resected stage III and IV thymoma and thymic carcinoma [4]. We consider that surgery is recommended for completely resectable thymoma with a few lung lesions. We do not perform adjuvant chemotherapy or radiotherapy in patients with totally resected stage III and IV thymoma and thymic carcinoma. After resection, surveillance for recurrence should include annual chest CT scan for at least ten years.

The second case was cytologically malignant; hence the type B3 thymoma produced a lung metastasis even after the initial surgery combined with adjuvant radiotherapy. There is still no standard guideline for the management of recurrent pulmonary metastases. Recurrence rates after resection of thymomas range from 5 to 50% and are related to the initial clinical stage [5]. The pattern of recurrence of resected thymomas includes local recurrence, pleural implants, and distant metastases. Local and distant recurrences occur with similar frequencies irrespective of the stage of the initial thymoma [2]. Complete resection of the local recurrent lesions seemed to be more effective for long-term survival [2,3,6]; likewise, complete resection of the recurrent pulmonary metastases may also result in a good prognosis [7]. We attempt surgery for lung metastases after the initial total thymectomy if the lesions appear completely resectable; whereas, it is necessary for us to discuss the indication for recurrent lung metastases after initial lung metastasectomy. In our opinion, types B3 and C thymomas have to be considered for re-resection because of higher risk of relapse. Alternative therapies (chemotherapy and radiotherapy) should be considered in the unresectable and the medically inoperable cases.

Long-term follow-up for thymoma patients is essential because thymomas are potentially malignant tumors.

## Conclusions

Here, we have reported two cases of metastasis from thymomas and reviewed the current literature. Surgery is the recommended option for a good prognosis for limited stage IVb patients if the thymomas with lung metastases appear completely resectable.

We emphasize the need for careful long-term follow-up of the patients who have undergone surgery to detect recurrences as early as possible. Complete resection of small numbers of recurrent pulmonary metastases may also result in a good prognosis.

## Consents

Written informed consent was obtained from the patients for publication of this case report and any accompanying images. A copy of the written consent is available for review by the Editor-in-Chief of this journal.

## Abbreviations

CT: computed tomography; FDG: ^18^ F-fluorodeoxyglucose; PET-CT: positron emission tomography-computed tomography; WHO: World Health Organization.

## Competing interests

The authors declare that they have no competing interest.

## Authors’ contributions

MK carried out the CT examinations and drafted the manuscripts the two cases. MH, MK, and DK performed the operation on Case 1. MN and DK performed the initial operation on Case 2, and MK, DK and YT performed the second operation. MK has followed-up the operations. All authors read and approved the final manuscript.

## References

[B1] Engels EA Pfeiffer RM Malignant thymoma in the United States: demographic patterns in incidence and associations with subsequent malignancies Int J Cancer 2003 105 546 551 10.1002/ijc.11099 12712448

[B2] Ruffini E Mancuso M Oliaro A Casadio C Cavallo A Cianci R Filosso PL Molinatti M Porrello C Cappello N Maggi G Recurrence of thymoma: analysis of clinicopathologic features, treatment, and outcome J Thorac Cardiovasc Surg 1997 113 55 63 10.1016/S0022-5223(97)70399-4 9011702

[B3] Detterbeck F Youssef S Ruffini E Okumura M A review of prognostic factors in thymic malignancies J Thorac Oncol 2011 6 S1698 S1704 21847050 10.1097/JTO.0b013e31821e7b12

[B4] Kondo K Monden Y Therapy for thymic epithelial tumors: a clinical study of 1,320 patients from Japan Ann Thorac Surg 2003 76 878 885 10.1016/S0003-4975(03)00555-1 12963221

[B5] Lucchi M Mussi A Surgical treatment of recurrent thymomas J Thorac Oncol 2010 5 S348 S351 20859131 10.1097/JTO.0b013e3181f20f27

[B6] Bae M Byun C Lee C Lee J Park I Kim D Yang W Chung K Clinical outcomes and prognosis of recurrent thymoma management J Thorac Oncol 2012 7 1304 1314 10.1097/JTO.0b013e3182598a91 22699889

[B7] Nomori H Watanabe K Ohtsuka T Naruke T Suemasu K Orikasa H Yamazaki K Pulmonary metastasis 12 years after resection of thymoma with microscopic capsule invasion Jpn J Ckin Oncol 2004 34 630 633 10.1093/jjco/hyh105 15591463

